# Age and Gender Differences in Physical Capability Levels from Mid-Life Onwards: The Harmonisation and Meta-Analysis of Data from Eight UK Cohort Studies

**DOI:** 10.1371/journal.pone.0027899

**Published:** 2011-11-16

**Authors:** Rachel Cooper, Rebecca Hardy, Avan Aihie Sayer, Yoav Ben-Shlomo, Kate Birnie, Cyrus Cooper, Leone Craig, Ian J. Deary, Panayotes Demakakos, John Gallacher, Geraldine McNeill, Richard M. Martin, John M. Starr, Andrew Steptoe, Diana Kuh, on behalf of the HALCyon study team

**Affiliations:** 1 MRC Unit for Lifelong Health and Ageing, Division of Population Health, University College London, London, United Kingdom; 2 MRC Lifecourse Epidemiology Unit, University of Southampton, Southampton General Hospital, Southampton, United Kingdom; 3 School of Social and Community Based Medicine, University of Bristol, Bristol, United Kingdom; 4 National Institute for Health and Research Musculoskeletal Biomedical Research Unit, University of Oxford, Oxford, United Kingdom; 5 Population Health Section, University Medical School, University of Aberdeen, Aberdeen, United Kingdom; 6 Centre for Cognitive Ageing and Cognitive Epidemiology, Department of Psychology, University of Edinburgh, Edinburgh, United Kingdom; 7 Department of Epidemiology and Public Health, University College London, London, United Kingdom; 8 Department of Primary Care and Public Health, Cardiff University, Cardiff, United Kingdom; 9 Centre for Cognitive Ageing and Cognitive Epidemiology, Geriatric Medicine, University of Edinburgh, Edinburgh, United Kingdom; Universidad Europea de Madrid, Spain

## Abstract

Using data from eight UK cohorts participating in the Healthy Ageing across the Life Course (HALCyon) research programme, with ages at physical capability assessment ranging from 50 to 90+ years, we harmonised data on objective measures of physical capability (i.e. grip strength, chair rising ability, walking speed, timed get up and go, and standing balance performance) and investigated the cross-sectional age and gender differences in these measures. Levels of physical capability were generally lower in study participants of older ages, and men performed better than women (for example, results from meta-analyses (N = 14,213 (5 studies)), found that men had 12.62 kg (11.34, 13.90) higher grip strength than women after adjustment for age and body size), although for walking speed, this gender difference was attenuated after adjustment for body size. There was also evidence that the gender difference in grip strength diminished with increasing age,whereas the gender difference in walking speed widened (p<0.01 for interactions between age and gender in both cases). This study highlights not only the presence of age and gender differences in objective measures of physical capability but provides a demonstration that harmonisation of data from several large cohort studies is possible. These harmonised data are now being used within HALCyon to understand the lifetime social and biological determinants of physical capability and its changes with age.

## Introduction

In recent years there have been an increasing number of projects initiated which aim to draw together data from a range of population-based studies to undertake cross-study collaborative work. This is due to the growing recognition of the potential scientific benefits to be gained as well as increasing expectations of study funders that data will be shared [1], [2]. The benefits of combining data include increased statistical power and the opportunity to validate findings across studies in a coordinated way. The latter can have the additional benefit of improving causal inference if the study populations examined have different confounding structures [3]–[9]. One of the main drivers of this trend has been the rise of genetic epidemiology which requires much larger sample sizes in order to achieve sufficient statistical power than analyses of traditional epidemiological risk factors [7]. However, this cross-study approach has applications and potential benefits across many areas of epidemiological research [10], including the study of ageing [6], [11], [12].

The Healthy Ageing across the Life Course (HALCyon) research programme is one example of a cross-study project on ageing. This interdisciplinary collaborative programme aims to identify how healthy ageing is affected by factors operating across life using data from nine UK cohort studies. It not only responds to recent calls for a life course approach to the epidemiological study of ageing [13], [14] but also provides an opportunity to investigate the challenges of harmonising data on ageing phenotypes across cohorts. Physical capability, a term used to describe an individual's ability to undertake the physical tasks of everyday living, is one of the main outcomes under investigation and the majority of analyses utilise the objective measures of physical capability that were available in these cohorts i.e. grip strength, chair rising ability, walking speed, timed get up and go and standing balance performance. By measuring physical performance and strength using objective tests, which not only indicate current physical capability levels but also predict future health and survival [15], [16], it is possible to examine variations in functioning across the whole spectrum of ability [17], in contrast to a focus on people at one extreme of the distribution with poor function or specific chronic conditions.

Associations between lower physical capability levels and higher mortality rates are consistently found [15], yet women have a longer average life expectancy than men despite having lower levels of physical capability and greater levels of self-reported functional limitations than men [18]–[28]. In addition to gender differences, age-related changes in physical capability are also well documented with consistent evidence of declining physical capability levels with increasing age shown in cross-sectional and longitudinal studies [20]–[23], [25], [26], [29]–[44]. Far fewer studies have examined whether gender differences in physical capability change with age and the majority of those that do focus on muscle strength. Most of these studies report gender differences in the average age at onset and/or rate of age-related decline in strength [20]–[22], [24], [25], [34], [35], [43], [45], and while the majority report greater rates of decline in strength with age among men than women [20], [21], [24], [25], [33], [35], [44], [46], a few studies report the reverse [26], [31]. Further study of age and gender differences in physical capability levels in UK cohorts is warranted as many existing studies have relatively small sample sizes and few studies cover the entire range of older ages, include a full range of objective measures of physical capability or include the British population.

By utilising data from 8 of the 9 UK cohorts participating in HALCyon, with ages at physical capability assessment ranging from 50 to 90+ years, we illustrate how the physical capability data were harmonised and then investigate the cross-sectional age and gender differences in these measures including an examination of age and gender interactions.

## Methods

### The HALCyon cohorts

Nine UK cohort studies, including approximately 40,000 individuals, contribute data to the HALCyon programme: the Lothian birth cohort 1921 (LBC1921); the Hertfordshire Ageing Study (HAS); the Hertfordshire Cohort Study (HCS); the Caerphilly Prospective Study (CaPS); the Boyd Orr cohort; the Aberdeen birth cohort 1936 (ABC1936); the English Longitudinal Study of Ageing (ELSA); the MRC National Survey of Health and Development (NSHD); and the National Child Development Study (NCDS). All cohorts, with the exception of ELSA and CaPS, have prospectively collected data from different stages of life enabling us to test life course hypotheses using cross-cohort comparative methods. ELSA and CaPS have been included because they have regularly obtained repeat measures of interest across later adult life. Full details of each of these studies are reported elsewhere [47]–[57] and are summarised in tables 1 and S1, figure S1 and below. Relevant ethical approval has been received for all studies.

**Table 1:** Summary of HALCyon cohorts.

	LBC1921	HAS	HCS	CaPS	Boyd Orr	ABC1936	ELSA	NSHD	NCDS
Year/s of birth	1921	1920–1930	1931–1939	1920–1939	1918–1939	1936	1912*–1952	1946	1958
**N** †	550	700 (1^st^ grip measure); 300 (second wave, all measures)	2997	1200	405	500	11,391 (core members total) 7700 with capability measures	2800	9,000
**Age/s at 1^st^ contact (y)**	11	Birth – 5	Birth – 5	45 – 59 (phase I)	0 – 19	11	50 – 90+	Birth	Birth
**Age/s (y) (Years) of subsequent contacts**	77–80 (1999–2001); 82–84 (2004); 85–87 (2007–2008)	63–73 (1994–5); 72–83 (2003–5)	59–73 (1999–2004); 65–74 (2004–5, E Herts only)	II: 47–67 (1984–8); III: 52–72 (1989–93); IV: 56–75 (1993–6); V: 65–84 (2002–4)	Flagged with NHS central register for vital status since 1948; questionnaire at ages 58–80 (1997–8); subsample at research clinic63–82 (2002–3)	62–68 (1999–2005)	52–90+ (2004 (wave 2)); 54–90+ (2006 (wave 3)) 56–90+ (2008 (wave 4))	Throughout childhood and adulthood (up to age 53y)	Throughout childhood and adulthood (up to age 44–5y)
**% women**	58.2	42.6	47.3	0	55.1	51.6	54.5	50.7	51.3
**% men**	41.8	57.4	52.7	100	44.9	48.4	45.5	49.3	48.7
**Age/s (y) at physical capability assessment**	77–80, 82–84 and 85–87	63–73 (grip) and 72–83 (all measures)	59–73 (first grip); 64–74 (second grip);62–74 (for all other measures)	65–84 (phase V)	63–82 (2002–3 clinic assessment)	62–68	Walking speed measured at all waves (60–90+); All other measures at w2 (52–90+) and 4 (56–90+)	53	Not assessed
**Physical capability measures assessed**	Grip strength at all 3 waves, walking speed at first and third wave	Grip strength at both ages; Chair rising; Walking speed; Timed get up and go; Standing balance (2nd age only)	Grip strength; Chair rising; Walking speed; Timed get up and go; Standing balance	Timed get up and go; Standing balance	Timed get up and go; Standing balance	Standing balance; Walking speed	Grip strength; Chair rising; Standing balance; Walking speed	Grip strength; Chair rising; Standing balance	n/a

*For those participants with a date of birth provided (95 participants were aged 90 or over at baseline and their dates of birth are not recorded).

†Approximate size of sample at time that outcome measures for the HALCyon project were ascertained.

Note: LBC1921  =  Lothian Birth Cohort 1921; HAS  =  Hertfordshire Ageing Study; HCS  =  Hertfordshire Cohort Study; CaPS  =  Caerphilly Prospective Study; ABC1936  =  Aberdeen Birth Cohort 1936; ELSA  =  English Longitudinal Study of Ageing; NSHD  =  MRC National Survey of Health and Development (1946 British birth cohort); NCDS  =  National Child Development Study (1958 British birth cohort).

For description of sample please see table S1.

#### The Lothian Birth cohort 1921 (LBC1921)

In 1932 a Scottish Mental Survey was administered to 11 y old school pupils (born in 1921) across Scotland. Members of this cohort were traced in the late 1990s and those still resident in the Lothian area of Scotland were recruited to participate in LBC1921 with the first wave of new data collected in 1999–2001 at an approximate age of 79 y [47].

#### Hertfordshire Ageing Study (HAS) and Hertfordshire Cohort Study (HCS)

HAS is a cohort of men and women born in North Hertfordshire, England, between 1920 and 1930 whose detailed birth and infant records were available and who were alive and still living in North Hertfordshire in 1994–5, aged 63–73 y, when the first new wave of data were collected [50]. HCS is a larger, younger cohort (born between 1931 and 1939), but similarly to HAS it consists of men and women born in Hertfordshire (East, North or West) whose birth and infant records were available and who were alive and still living in Hertfordshire in the 1990s. The first new wave of data for HCS were collected in 1999–2004 when study participants were aged 59–73 y [49].

#### Caerphilly Prospective Study (CaPS)

CaPS is a cohort of men, born between 1920 and 1939, who were recruited when they were aged 45–59 years, between 1979 and 1983, from the town of Caerphilly and adjacent villages in South Wales [48]. During the second wave, between 1984 and 1988, the original cohort was supplemented with men of a similar age who had moved into the defined area.

#### Boyd Orr Cohort

The Boyd Orr cohort consists of men and women born between 1918 and 1939 who participated in the Carnegie (Boyd Orr) Survey of Diet and Health in Pre-War Britain, 1937–1939. In the original survey, 4,999 boys and girls aged 0–19 y in 16 centres across the UK underwent a range of assessments. In 1997, a follow-up study re-established contact with participants of the survey using the National Health Service Central Register and its Scottish equivalent in Edinburgh. At this time 3,182 participants from the original sample were traced, alive and residing in the UK. These participants were sent a detailed health and lifestyle questionnaire to which 1,648 responded. In 2002, all 732 surviving study members aged 63–83 y who lived near clinics in Bristol, London, Wisbech, Aberdeen and Dundee, and had previously consented to clinical follow-up were contacted of whom 405 participated in a detailed clinical examination and questionnaire [51], [52].

#### Aberdeen Birth Cohort 1936 (ABC1936)

ABC1936 is a cohort of men and women born in 1936 who participated in the Scottish Mental Survey administered to 11 y old school pupils across Scotland in 1947. The cohort were traced in the late 1990s and those still resident in the Aberdeen area of Scotland were recruited to participate in ABC1936 with the first wave of new data collected between ages 62 and 68 y [47].

#### English Longitudinal Study of Ageing (ELSA)

ELSA is a cohort of men and women, who were born between the early 1900s and February 1952 and were sampled from private households in England that had previously participated in the Health Survey for England in 1998, 1999 or 2001. Since the first ELSA wave in 2002, the cohort have been followed up every two years and while additional participants have been added at subsequent waves these analyses utilise data only on the study participants who were included at baseline and wave 1 [53]–[55].

#### MRC National Survey of Health and Development (NSHD) and the National Child Development Study (NCDS)

The NSHD, alternatively known as the 1946 British birth cohort, is a nationally representative sample of people born in England, Scotland and Wales during one week in March 1946 who have been followed up prospectively since birth [56]. Similarly to NSHD, NCDS is also a British birth cohort, alternatively known as the 1958 British birth cohort, and consists of a nationally representative sample of people born in England, Scotland and Wales who have been followed up prospectively since birth in March 1958 [57].

### Ascertainment and harmonisation of physical capability measures

With the exception of NCDS, all the cohorts contributing data to HALCyon have measured physical capability levels objectively during at least one wave in mid-life or later adulthood (tables 1 and S2).

#### Grip strength

Grip strength has been assessed in LBC1921, HAS, HCS, ELSA and NSHD. During the first wave of HAS Harpenden handgrip dynamometers were used to measure grip strength. In LBC1921, HCS and the second wave of HAS, grip strength measurements were taken using a Jamar hydraulic dynamometer (or North Coast Hydraulic Hand dynamometer in some LBC1921 participants) with three measures in the dominant hand recorded in LBC1921 and three measures in each hand recorded in HCS and HAS. In ELSA three measures in each hand were taken using a Smedley's handgrip dynamometer and in NSHD a Nottingham electronic handgrip dynamometer [58], [59] was used to record two measures in each hand. In all five cohorts the maximum grip strength (kg) achieved was used in the main analyses, with some analyses also rerun after standardisation to help account for differences between studies in absolute levels of strength recorded due to differences in dynamometer type.

#### Chair rises

Chair rising ability has been assessed in HAS, HCS, ABC1936, ELSA and NSHD. In HAS, HCS and all ELSA participants the time taken for study participants to rise from a sitting to a standing position and sit down again five times, as fast as possible, was recorded. In the NSHD and among ELSA participants aged 69 and under, the same test was undertaken but 10 rises were performed. In ABC1936 the ability to stand from a sitting position was assessed but chair rise time was not recorded and so ABC1936 has not been included in these analyses.

For the purposes of comparability across cohorts the time taken to rise from a chair five times was estimated in the NSHD. This was done using data from ELSA participants who had times recorded for both 10 and 5 chair rises and who were of a similar age to NSHD participants. By regressing the time taken to rise from a chair five times on the time taken to rise from a chair ten times for ELSA participants, a regression equation was obtained to predict a time for five chair rises from the recorded time for 10 rises in the NSHD. As the distributions of chair rise times tend to be skewed the times for five chair rises in each of the cohorts underwent natural log transformations.

#### Walking speed

Walking speed has been assessed in LBC1921, HAS, HCS, ABC1936 and ELSA. In LBC1921 the time taken to walk 6 m as quickly as possible was recorded. In all other cohorts the time taken to walk at a normal pace distances of 3 m (HAS and HCS), 6 m (ABC1936) and 8 feet (ELSA) from a standing start were recorded, with two trials of this test performed in ELSA.

To ensure comparability of measures across cohorts walking speeds in metres/second were calculated by dividing the distance walked in metres by the time recorded. By converting the times to speeds the distribution is also more normally distributed. In ELSA the average speed of the two trials was calculated.

#### Timed Get Up and Go (TUG)

In four of the cohorts, HAS, HCS, CaPS and Boyd Orr, the time to get up from a chair, walk 3 m at a normal pace, turn around, return to the chair and sit back down again (with or without use of a walking aid) had been recorded [60]. In CaPS and Boyd Orr two trials were performed. Speed was calculated, for comparison with walking speeds and to normalise the distributions, by dividing 6 (i.e. the distance in metres walked) by the times. The average speeds for the two trials performed were calculated for CaPS and Boyd Orr.

#### Standing balance

Standing balance performance has been assessed in HAS, HCS, CaPS, Boyd Orr, ABC1936, ELSA and NSHD. In HAS, HCS, CaPS, Boyd Orr and NSHD the longest time up to 30 seconds that a one-legged stand could be maintained with eyes open was recorded, with two trials performed in CaPS and Boyd Orr and one trial in all other cohorts. In the NSHD, this test was also repeated with eyes closed. In ABC1936 whether participants were able to balance on one leg with their eyes open for 5 s was recorded. In ELSA, a series of stands were performed, with participants first asked to stand side by side for 10 seconds, then in a semi-tandem stand for 10 s and finally in a full tandem stand for 10 s, with participants only completing the next stand if they were able to complete the full 10 s of the previous stand. The time, up to a maximum of 30 s, that all participants aged 69 and under could balance on one leg with eyes open was assessed if completion of all three stages of the tandem stands were successful. However, this measure was not used as it would have meant the exclusion of all ELSA participants aged 70+.

As the distributions of balance times were severely skewed, there was a ceiling effect at 30 s in those cohorts that had measured times up to 30 s and to enable inclusion of ABC1936 and ELSA, binary variables for standing balance were created. These indicated whether or not an individual was able to balance on one leg with their eyes open for at least 5 seconds. In Boyd Orr and CaPS the best of the two times recorded was used. In ELSA those people unable to perform the side by side and semi-tandem stands for 10 s were categorised as unable and those people who performed the full tandem stand were categorised according to whether their time was below or above 5 seconds.

#### Repeat measures

As the analyses undertaken were cross-sectional, where physical capability had been assessed at more than one wave within a cohort (as shown in table 1) the majority of analyses presented utilise measures from the first recorded wave to maximise sample size. There are two exceptions to this. Firstly, for walking speed in ELSA our main analyses utilise the measure from wave 2, given all other physical capability measures in this cohort were not assessed for the first time until this wave. Secondly, in HCS, for all physical capability measures with the exception of grip strength, some study participants were assessed during the 1999–2004 wave while others were assessed during the 2004–2005 wave. To maximise the sample size, we therefore combined the measures from the two waves taking the measure from the first wave if available and from the second wave if not available at the first but available at the second (with no overlap in participants between the waves for chair rises and standing balance and with only 135 participants having completed tests of walking speed and TUG at both waves).

### Analyses

We calculated descriptive statistics for each physical capability measure by gender and 5-year age group within each cohort. Tests for trend across age groups were performed in cohorts where participants' ages spanned more than one 5-year age group. Age-adjusted gender differences in each physical capability measure in each cohort, except CaPS which only includes men, were then tested using regression models (logistic regression for standing balance and linear regression for all other measures). Random effects meta-analyses (selected *a priori* due to expected heterogeneity) of the regression coefficients were performed to produce overall summary estimates of the age-adjusted gender differences in each of the five physical capability measures. These analyses were then repeated with additional adjustment for current body size, due to the gender differences in body size and the expected influence of weight and height on physical capability, using weight (kg) and height (cm) where available and body mass index and height in Boyd Orr. Between-study heterogeneity was investigated using I^2^ and *Q* statistics [61], [62].

To test whether gender differences in physical capability changed with age we then tested, in those cohorts with sufficient variation in gender and age (hence excluding LBC1921 and NSHD), interactions between gender and age. Again, these analyses were performed separately within each cohort before performing random effects meta-analyses to calculate the overall summary estimates [63]. Models were fitted including the main effects of gender and age (centred at 60 years) and the interaction between the two. The analyses were then repeated with adjustment for current body size. All analyses were rerun using measures of physical capability which had been standardised by gender and cohort to assess the impact of gender and cohort differences in the distributions of the physical capability measures.

In those cohorts where those participants who were unable to perform each of the physical capability tests could be identified, chi-square tests were undertaken to compare age and gender differences among those able to perform the tests with those unable to perform the tests who were excluded from the main analyses.

## Results

Within cohorts, younger participants tended to have higher levels of physical capability as indicated by stronger grip strength, shorter chair rise times (table 2), faster walking and TUG speeds (table 3) and lower odds of inability to balance for 5 seconds (table 4) than older participants (p<0.01 in the majority of tests for trend across 5 year age groups) (tables 2, 3, and 4).

**Table 2:** Descriptive statistics for grip strength and chair rise time by age group and gender in each HALCyon cohort.

		Total										
		N*	Age range	50–54^a^	55–59^b^	60–64^c^	65–69	70–74^d^	75–79^e^	80–84^f^	85–89	90+
**Grip strength (kg)**				**Mean (sd)**								
**LBC1921**	**M**	229	77–80	-	-	-	-	-	34.71 (7.4)	-	-	-
	**W**	315		-	-	-	-	-	20.60 (4.5)	-	-	-
**HAS**	**M**	411	63–73	-	-	40.74 (5.9)	37.84 (7.6)	37.01 (5.8)	-	-	-	-
	**W**	305				24.08 (5.8)	22.67 (5.1)	20.89 (5.5)	-	-	-	-
**HCS**	**M**	1572	59–73	-	49.31 (4.7)	45.31 (7.2)	43.29 (7.6)	41.94 (7.2)	-	-	-	-
	**W**	1415		-	-	27.27 (5.8)	26.44 (5.7)	25.01 (5.5)	-	-	-	-
**ELSA**	**M**	3426	52–90+	48.21 (7.9)	45.69 (8.5)	42.86 (9.1)	40.16 (8.3)	37.94 (7.4)	34.01 (7.5)	30.23 (7.4)	28.12 (7.2)	26.19 (5.7)
	**W**	4127		28.38 (5.8)	27.19 (5.9)	25.76 (6.1)	24.54 (5.4)	22.57 (5.6)	20.55 (5.3)	17.94 (5.2)	17.22 (4.5)	14.59 (4.9)
**NSHD**	**M**	1406	53–53	47.64 (12.2)	-	-	-	-	-	-	-	-
	**W**	1444		27.76 (7.9)	-	-	-	-	-	-	-	-
**Chair rise time (s)**				**Median (IQR)**								
**HAS**	**M**	162	72–83	-	-	-	-	17.28 (13.5;21.1)	19.22 (16.9;23.4)	16.47 (12.9;21.5)	-	-
	**W**	110		-	-	-	-	20.76 (17.0;25.4)	20.08 (17.1;24.0)	18.50 (17.9;29.3)	-	-
**HCS**	**M**	647	62–74	-	-	14.53 (12.8;15.6)	14.78 (12.9;16.9)	15.52 (13.7;18.1)	-	-	-	-
	**W**	951		-	-	17.09 (14.4;21.0)	17.19 (14.7;20.2)	17.44 (14.3;20.7)	-	-	-	-
**ELSA**	**M**	2899	52–90+	9.36 (7.8;11.3)	9.68 (8.0;11.6)	10.04 (8.5;12.4)	10.53 (8.8;12.7)	11.84 (9.8;14.1)	12.83 (10.9;15.8)	14.00 (11.3;17.5)	14.48 (11.2;19.2)	18.72 (15.2;20.3)
	**W**	3435		9.47 (8.0;11.7)	9.82 (8.0;12.0)	10.46 (8.7;12.9)	11.12 (9.3;13.5)	12.59 (10.4;15.4)	13.45 (11.2;16.2)	14.18 (11.7;18.0)	15.27 (12.6;18.6)	16.05 (12.6;18.1)
**NSHD**	**M**	1344	53–53	9.71† (7.9;11.6)	-	-	-	-	-	-	-	-
	**W**	1394		9.43† (8.1;11.6)	-	-	-	-	-	-	-	-

Note: LBC1921  =  Lothian Birth Cohort 1921; HAS  =  Hertfordshire Ageing Study; HCS  =  Hertfordshire Cohort Study; ELSA  =  English Longitudinal Study of Ageing; NSHD  =  MRC National Survey of Health and Development (1946 British birth cohort).

a : 53 y only in NSHD; 52–54 y in ELSA;

b : 59 y only in HCS;

c : 63–64 y in HAS; 62–64 y in HCS for chair rises;

d : 70–73 y in both HAS and HCS for grip strength; 72–74 y in HAS for chair rises;

e : 77–80 y in LBC1921;

f : 80–83 y in HAS.

†Estimated time for completion of 5 chair rises (for comparison with other cohorts).

*Approximate number of men (M) and women (W) study participants in each age category (Ns vary slightly between different capability measures and so those presented are for the sample with data on at least one of the capability measures unless the different capability measures have been assessed in different waves or on slightly different sub-samples):

HAS (grip strength): 63–64 y: M = 79, W = 40; 65–69 y: M = 264, W = 211; 70–74 y: M = 68, W = 54

HAS (chair rises): 72–74 y: M = 58, W = 36; 75–79 y: M = 106, W = 81; 80–83 y: M = 9, W = 4

HCS (grip strength): 59 y: M = 13, W = 0; 60–64 y: M = 642, W = 426; 65–69 y: M = 779, W = 809; 70–73 y: M = 138, W = 180

HCS (chair rises): 62–64 y: M = 53, W = 138; 65–69 y: M = 386, W = 543; 70–74 y: M = 208, W = 270

ELSA: 52–54 y: M = 291, W = 333; 55–59 y: M = 759, W = 906; 60–64 y: M = 577, W = 713; 65–69 y: M = 595, W = 678; 70–74 y: M = 507, W = 556; 75–79 y: M = 367, W = 461; 80–84 y: M = 233, W = 352; 85–89 y: M = 94, W = 164; 90+y: M = 28, W = 52.

**Table 3:** Descriptive statistics for walking and timed get up and go (TUG) speed by age group and gender in each HALCyon cohort.

		Total								
		N*	Age range	60–64^a^	65–69^b^	70–74^c^	75–79^d^	80–84^e^	85-89	90+
**Walking speed (m/s)**				**Mean (sd)**						
**LBC1921**	**M**	229	77–80	-	-	-	1.50 (0.4)	-	-	-
	**W**	312		-	-	-	1.31 (0.3)	-	-	-
**HAS**	**M**	170	72-83	-	-	0.93 (0.2)	0.84 (0.2)	0.79 (0.2)	-	-
	**W**	119		-	-	0.80 (0.2)	0.79 (0.2)	0.43 (0.1)	-	-
**HCS**	**M**	1087	61–74	0.97 (0.1)	0.94 (0.2)	0.91 (0.2)	-	-	-	-
	**W**	1213		0.95 (0.2)	0.91 (0.2)	0.87 (0.2)	-	-	-	-
**ABC1936**	**M**	240	62–68	1.24 (0.3)	1.29 (0.3)	-	-	-	-	-
	**W**	254		1.20 (0.2)	1.20 (0.2)	-	-	-	-	-
**ELSA M**	**M**	2454	60–90+	1.00 (0.3)	0.95 (0.3)	0.88 (0.3)	0.81 (0.2)	0.72 (0.3)	0.63 (0.2)	0.56 (0.3)
	**W**	3045		0.95 (0.3)	0.91 (0.3)	0.82 (0.3)	0.74 (0.2)	0.62 (0.2)	0.55 (0.2)	0.47 (0.2)
**TUG speed (m/s)**				**Mean (sd)**						
**HAS**	**M**	172	72–83	-	-	0.57 (0.1)	0.49 (0.1)	0.49 (0.2)	-	-
	**W**	120		-	-	0.48 (0.1)	0.48 (0.1)	0.32 (0.1)	-	-
**HCS**	**M**	1090	61–74	0.59 (0.1)	0.58 (0.1)	0.56 (0.1)	-	-	-	-
	**W**	1216		0.58 (0.1)	0.56 (0.1)	0.54 (0.1)	-	-	-	-
**CaPS**	**M**	1114	65–84	-	0.62 (0.1)	0.58 (0.1)	0.55 (0.1)	0.48 (0.1)	-	-
**Boyd Orr**	**W**	182	63–82	0.71 (0.1)	0.67 (0.1)	0.64 (0.2)	0.56 (0.1)	0.58 (0.1)	-	-
	**M**	223		0.71 (0.1)	0.67 (0.1)	0.63 (0.1)	0.56 (0.2)	0.48 (0.2)	-	-

Note: LBC1921  =  Lothian Birth Cohort 1921; HAS  =  Hertfordshire Ageing Study; HCS  =  Hertfordshire Cohort Study; CaPS  =  Caerphilly Prospective Study; ABC1936  =  Aberdeen Birth Cohort 1936; ELSA  =  English Longitudinal Study of Ageing.

a : 63–64 y in HAS and Boyd Orr; 61–64 y in HCS;

b : 65–68 y in ABC1936;

c : 72–74 y in HAS;

d : 77–80 y in LBC1921;

e : 80–83 y in HAS; 80–82 y in Boyd Orr.

*Approximate number of men (M) and women (W) study participants in each age category (Ns vary slightly between different capability measures and so those presented are for the sample with data on at least one of the capability measures unless the different capability measures have been assessed in different waves or on slightly different sub-samples):

HAS: 72–74 y: M = 58, W = 36; 75–79 y: M = 106, W = 81; 80–83 y: M = 9, W = 4.

HCS: 61–64 y: M = 201, W = 257; 65–69 y: M = 630, W = 705; 70–74 y: M = 259, W = 254.

ELSA: 60–64 y: M = 606, W = 744; 65–69 y: M = 600, W = 689; 70–74 y: M = 508, W = 587; 75–79 y: M = 395, W = 481; 80–84 y: M = 227, W = 351; 85–89 y: M = 89, W = 148; 90+y: M = 29, W = 45.

ABC1936: 62–64 y: M = 144, W = 161; 65–68 y: M = 100, W = 99.

CaPS: 65–69 y: M = 307; 70–74 y: M = 440; 75–79 y: M = 325; 80+y: M = 79.

Boyd Orr: 63–64 y: M = 11, W = 17; 65–69 y: M = 69, W = 98; 70–74 y: M = 72, W = 68; 75–79 y: M = 26, W = 37; 80–82 y: M = 4, W = 3.

**Table 4:** Descriptive statistics for standing balance by age group and gender in each HALCyon cohort.

		Total										
		N*	Age range	50–54^a^	55–59	60–64^b^	65–69^c^	70–74^d^	75–79	80–84^e^	85-89	90+
**Standing balance**				**% unable to balance for at least 5 seconds** §								
**HAS**	**M**	173	72–83	-	-	-	-	20.69	37.74	22.22	-	-
	**W**	121		-	-	-	-	20.56	38.27	75.00	-	-
**HCS**	**M**	669	62–74	-	-	8.93	15.29	21.96	-	-	-	-
	**W**	986		-	-	16.31	16.01	27.21	-	-	-	-
**CaPS**	**M**	1123	65–84	-	-	-	13.29	22.48	36.01	53.33	-	-
**Boyd Orr**	**W**	181	63–82	-	-	9.09	14.49	19.72	26.92	50.00	-	-
	**M**	223				5.88	13.27	20.59	45.95	66.67	-	-
**ABC1936**	**W**	200	62–68	-	-	3.00	10.00	-	-	-	-	-
	**M**	211		-	-	4.46	13.13	-	-	-	-	-
**ELSA**	**W**	3451	52–90+	3.09	4.48	6.24	9.75	13.21	20.98	34.33	55.32	64.29
	**M**	4215		3.30	5.41	7.99	11.80	23.02	30.80	53.98	63.41	92.31
**NSHD**	**W**	1415	53–53	3.53	-	-	-	-	-	-	-	-
	**M**	1463		5.33	-	-	-	-	-	-	-	-

Note: HAS  =  Hertfordshire Ageing Study; HCS  =  Hertfordshire Cohort Study; CaPS  =  Caerphilly Prospective Study; ABC1936  =  Aberdeen Birth Cohort 1936; ELSA  =  English Longitudinal Study of Ageing; NSHD  =  MRC National Survey of Health and Development (1946 British birth cohort).

a : 53 y only in NSHD; 52–54 y in ELSA;

b : 63–64 y in HAS and Boyd Orr; 62–64 y in ABC1936 and in HCS;

c : 65–68 y in ABC1936;

d : 72–74 y in HAS;

e : 80–83 y in HAS; 80–82 y in Boyd Orr.

§Assessed with eyes open.

*Approximate number of men (M) and women (W) study participants in each age category (Ns vary slightly between different capability measures and so those presented are for the sample with data on at least one of the capability measures unless the different capability measures have been assessed in different waves or on slightly different sub-samples):

HAS: 72–74 y: M = 58, W = 36; 75–79 y: M = 106, W = 81; 80–83 y: M = 9, W = 4.

HCS: 62–64 y: M = 56, W = 141; 65–69 y: M = 399, W = 562; 70–74 y: M = 214, W = 283.

ELSA: 52–54 y: M = 291, W = 333; 55–59 y: M = 759, W = 906; 60–64 y: M = 577, W = 713; 65–69 y: M = 595, W = 678; 70–74 y: M = 507, W = 556; 75–79 y: M = 367, W = 461; 80–84 y: M = 233, W = 352; 85–89 y: M = 94, W = 164; 90+y: M = 28, W = 52.

ABC1936: 62–64 y: M = 144, W = 161; 65–68 y: M = 100, W = 99.

CaPS: 65–69 y: M = 307; 70–74 y: M = 440; 75–79 y: M = 325; 80+y: M = 79.

Boyd Orr: 63–64 y: M = 11, W = 17; 65–69 y: M = 69, W = 98; 70–74 y: M = 72, W = 68; 75–79 y: M = 26, W = 37; 80–82 y: M = 4, W = 3.

**Figure 1:**
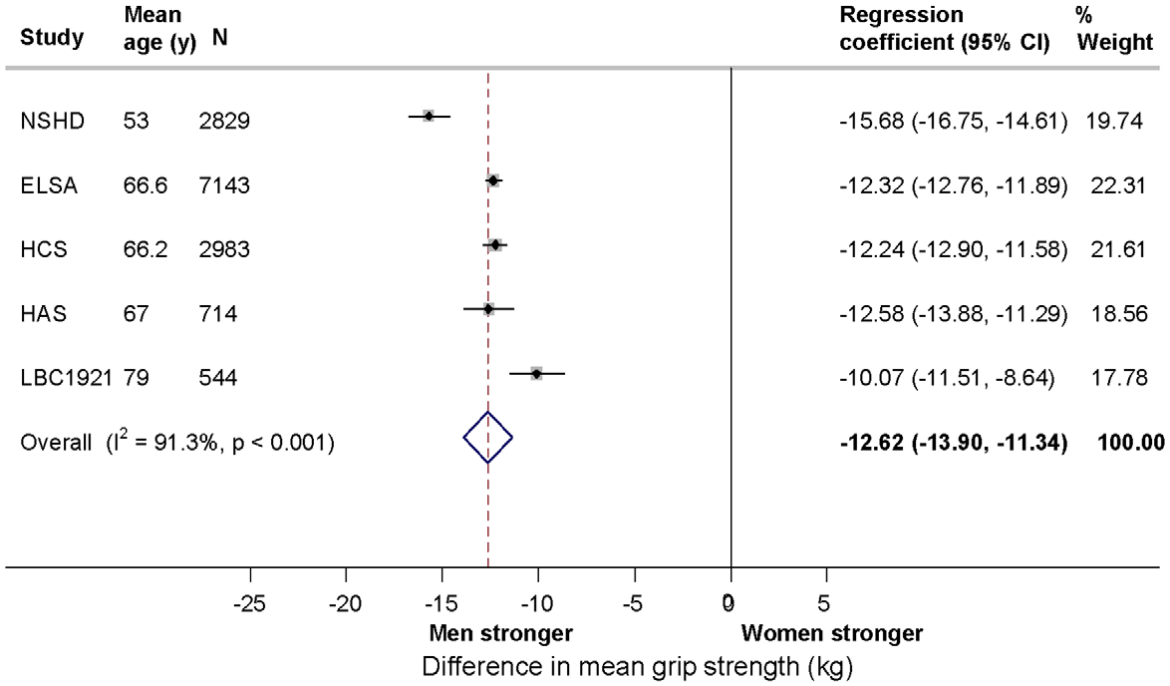
Gender differences in mean grip strength (kg) adjusted for age, height and weight in the HALCyon cohorts (comparing women with men (reference category)). The abbreviations of study names for figures 1–5 are: ABC1936: Aberdeen Birth Cohort 1936; Boyd Orr; CaPS: Caerphilly Prospective Study; ELSA: English Longitudinal Study of Ageing; HAS: Hertfordshire Aging Study; HCS: Hertfordshire Cohort Study; LBC1921: Lothian Birth Cohort 1921; NSHD: MRC National Survey of Health and Development.

Men had mean grip strength at least 10 kg greater than women, after adjustment for age and body size (figure 1). In meta-analyses (N = 14,213 (5 studies)), the overall summary estimates of the differences in mean grip strength between men and women were 16.67 kg (95% CI: 15.26, 18.08) after adjustment for age and, 12.62 kg (11.34, 13.90) after subsequent adjustment for body size (table 5). Although there was evidence in all cohorts of this gender difference, there was substantial heterogeneity between studies, I^2^  =  91.3% in adjusted analyses. This may be partially explained by variation in the size of the gender difference by age; the gender difference decreased with increasing mean age of study participants (figure 1). In meta-analyses of age by gender interaction terms (N = 10,840 (3 studies)) the overall summary effect was 0.25 (0.22, 0.28) (I^2^  =  0.0%) after adjustment for body size. The size and direction of this interaction term indicates that the gender difference diminished with increasing age. A similar interaction was also found when grip strength was standardised by gender and cohort, suggesting that changing gender differences in the distribution of grip strength with age do not explain the interaction (results not shown).

**Table 5:** Summary effect estimates from meta-analyses of the gender differences in physical capability across the HALCyon cohorts.

			Comparison of women with men (reference)					
	Number of:		Age-adjusted			Age and body size adjusted*		
	studies	participants	Regression coefficient‡ (95% CI)	I^2^ (%)	p†	Regression coefficient‡ (95% CI)	I^2^ (%)	p†
**Grip strength (kg)**	5	14,213	-16.67 (-18.08, -15.26)	96.4	<0.001	-12.62 (-13.90, -11.34)	91.3	<0.001
**Chair rise time (ln(s))**	4	10,754	0.07 (0.01, 0.13)	95.7	<0.001	0.11 (0.05, 0.17)	92.4	<0.001
**Walking speed (m/s)**	5	8,246	-0.08 (-0.11, -0.04)	87.9	<0.001	-0.02 (-0.06, 0.01)	78.4	0.001
**TUG speed (m/s)**	3	2,997	-0.02 (-0.04, -0.003)	54.7	0.11	-0.01 (-0.04, 0.02)	72.7	0.03
**Standing balance**	6	12,838	1.48 (1.27, 1.72)	23.3	0.26	1.34 (1.13, 1.59)	0.0	0.93

† p-values from Cochran's *Q* statistic.

‡Regression coefficients are the difference in means comparing women with men for grip strength, chair rise time, walking speed and TUG speed and odds ratio of inability to balance for 5 s with eyes open comparing women with men.

*adjusted for current height and weight, except for Boyd Orr where adjustment was made for body mass index and height.

Exclusion of LBC1921 from walking speed analyses (4 studies, 7705 participants): age-adjusted: -0.06 (-0.08, -0.03), I^2^  =  77.6%, p = 0.004; age and body size adjusted: -0.01 (-0.03, 0.02), I^2^  =  62.1%, p = 0.05.

**Figure 2:**
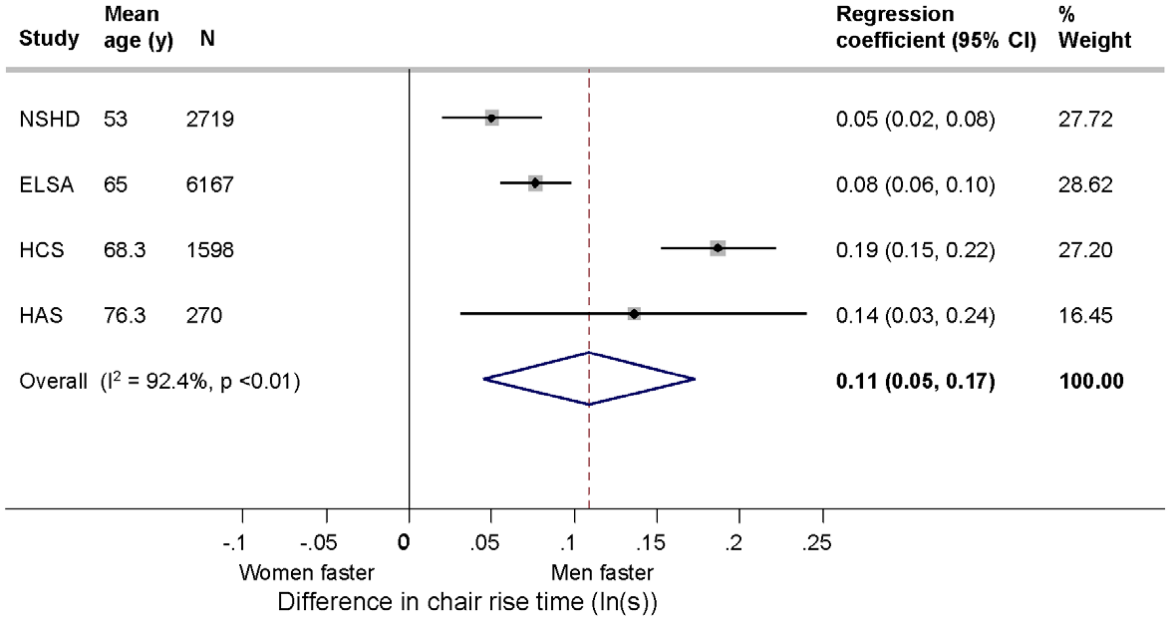
Gender differences in mean chair rise time (ln(s)) adjusted for age, height and weight in the HALCyon cohorts (comparing women with men (reference category)).

On average, men performed five chair rises faster than women (figure 2). The overall summary estimates of the differences in mean chair rise time (ln(s)) (N = 10,754 (4 studies)) when comparing women with men were 7% (95% CI: 1%, 13%) after age adjustment and, 11% (5%, 17%) after subsequent adjustment for body size (table 5). There was no suggestion that the heterogeneity (I^2^  = 92.4%) was explained by gender differences varying by age. This was supported by there being no evidence of an interaction between age and gender (((N = 8,035 (3 studies)) overall summary interaction term -0.01 (-0.02, 0.01)).

**Figure 3:**
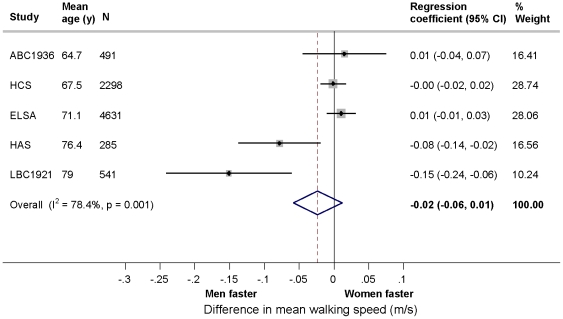
Gender differences in mean walking speed (m/s) adjusted for age, height and weight in the HALCyon cohorts (comparing women with men (reference category)).

Prior to adjustment for body size, there was evidence that men had faster walking speeds than women (table 5). After adjustment the gender difference was attenuated (figure 3). The overall summary estimates of the differences in mean walking speed (m/s) (N = 8,246 (5 studies)) when comparing women with men were -0.08 (95% CI: -0.11, -0.04) after age adjustment and, -0.02 (-0.06, 0.01) after subsequent adjustment for body size (table 5). Similar results were found when results from LBC1921, which measured fastest rather than normal walking pace, were excluded. In meta-analyses of the interaction between age and gender (N = 7,705 (4 studies)) there was weak evidence of this (overall summary interaction term -0.002 (-0.004, -0.0001)); when using either measure of walking speed (i.e. m/s or an SD score) (results not shown), the gender difference increased with increasing age.

**Figure 4:**
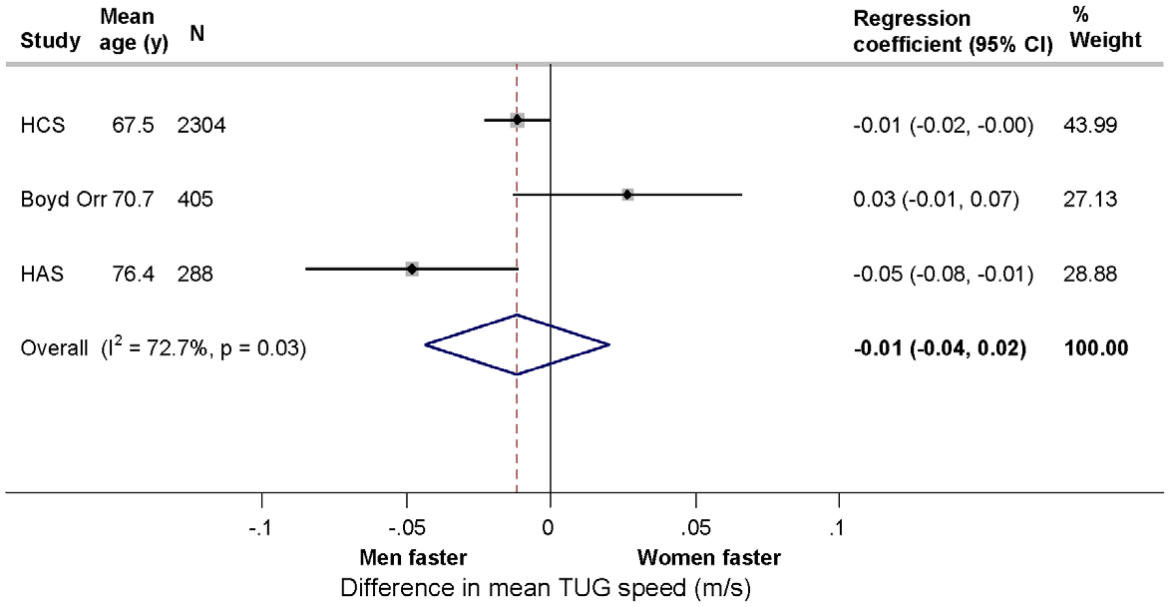
Gender differences in mean timed get up and go (TUG) speed (m/s) adjusted for age, height and weight in the HALCyon cohorts (comparing women with men (reference category)).

There was no clear evidence of a gender difference in TUG speed either prior to or after adjustment for body size (table 5 and figure 4) or of an interaction between age and gender.

**Figure 5:**
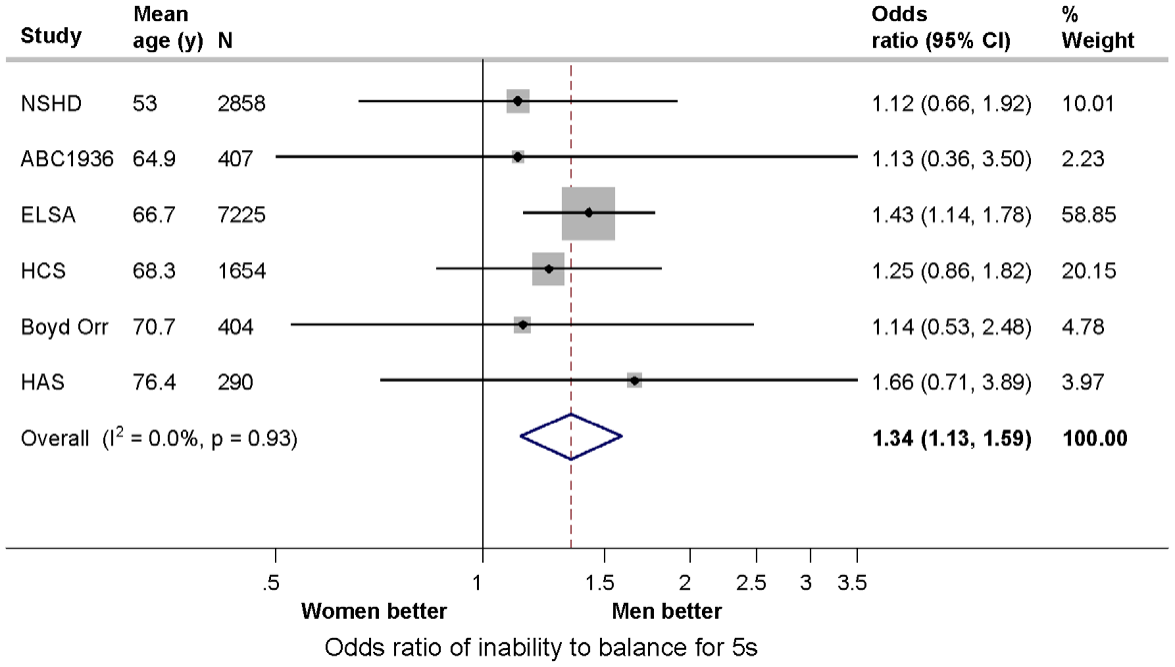
Odds ratios of inability to balance for 5 seconds comparing women with men adjusted for age, height and weight in the HALCyon cohorts.

Women were at increased odds of being unable to balance compared with men (figure 5). The overall summary odds ratios of inability to balance for 5 s (N = 12,838 (6 studies)), comparing women with men were 1.48 (95% CI: 1.27, 1.72) after age adjustment and 1.34 (1.13, 1.59) after additional adjustment for body size (table 5). This pattern of association was consistently found across studies (I^2^  =  0.0% in body size adjusted analyses). There was no evidence to suggest that this gender difference varied by age (((N = 9,980 (5 studies)) overall summary interaction term 1.02 (1.00, 1.03)).

There was some evidence that older participants were less likely to be able to complete the physical capability tests than younger participants (table S3). In ELSA there was also evidence that women were less likely than men to be able to perform the grip strength and chair rising assessments, but similar gender differences were not found in other cohorts.

## Discussion

### Main findings

There are age and gender differences in physical capability levels in UK cohorts born across the first half of the twentieth century, assessed at age 50 y and above; levels of physical capability decline with age and men perform better than women in most measures, although for walking speed this gender difference was attenuated after adjustment for body size. There was also evidence that the gender difference in grip strength diminished with increasing age and there may be a widening gender difference in walking speed with increasing age. These analyses also demonstrate that data harmonisation of physical capability measures is possible and that, while caution is still required, these data can be used in a coordinated way.

### Comparisons with other studies

These data are some of the first to be presented which demonstrate the nature of the age and gender differences in objective measures of physical capability across a large, representative sample of older British people. While absolute levels of physical capability vary between countries [23], [43], [64] our finding of age and gender differences in the majority of measures confirms that the patterns of these differences in the UK are similar to those in many other countries [20], [21], [23], [25], [26], [35]–[42].

Our observation of a diminishing gender difference in grip strength with increasing age is consistent with the majority of studies [20], [21], [24], [25], [33], [35], [44]–[46]. As women tend to have weaker strength than men, our finding is also consistent with studies showing that those people who have greater strength at baseline are more likely to experience a faster rate of strength loss with increasing age than those who are initially weaker [32], [46]. Few previous studies have assessed the interaction of age and gender in association with other physical capability measures but, in one American cross-sectional study [65] a faster decline in walking speed was found among women up to age 62 y whereas a faster decline was found in men thereafter.

### Explanations of findings

There are a number of factors which are likely to contribute to the finding of declining physical capability levels with increasing age. From mid-life onwards as individuals age their muscle mass declines usually as a result of a loss of muscle fibres and reductions in the volume of the remaining muscle fibres [66], [67]. While declining muscle mass is likely to impact on strength and hence also physical performance, strength has been found to decline more rapidly than mass [46] and muscle mass does not fully explain variability in muscle strength [45], [68]. Other characteristics of muscle which change with increasing age including declining muscle quality which includes increased denervation and fat infiltration are therefore also likely to be influential [66], [67], [69].

In addition to changes in the structure and function of muscle with age that impact on physical performance, either directly or acting through strength [70], changes with age in neurological function [68], cardiovascular function and fitness [71], hormonal exposures, body size and health behaviours including physical activity and diet [45] may impact on physical capability. As people age they are also more likely to develop chronic conditions that detrimentally impact on their physical capability.

As the differences in physical capability by age demonstrated in this study were based on cross-sectional data the possibility that these differences are not the result of longitudinal age-related changes must be considered. Secular increases in peak grip strength that are not fully explained by the secular increases in body size have recently been demonstrated [21]. However, secular trends seem unlikely to fully explain the age differences in physical capability found.

Gender differences in physical capability levels are likely to be partially explained by differences in body composition [72]. Due to genetic, hormonal and environmental differences men tend to have a higher proportion of lean mass than women. There are also gender differences in the distribution of lean mass with men tending to have greater amounts of upper body lean mass [72], which is particularly important when considering grip strength. Furthermore, women may be more likely than men to meet the definitions of both sarcopenia and obesity with the combined effects of reduced muscle mass and excessive body weight negatively impacting on physical capability. There are also gender differences in other factors which may impact on physical capability including health behaviours, self-perceptions of health, health care utilisation and risk of chronic conditions [18], [27], [28].

The changes in the size of the gender differences in grip strength and walking speed with age may be explained by gender differences in age-related changes in factors which impact on physical capability including physiological functions such as inflammation [73]. They may also be at least partially explained by gender and age differences in the prevalence of inability to perform the tests. In the two studies where gender differences in inability to perform grip strength measurements could be assessed, women were more likely to be unable than men (table S3) and risk of being unable to perform the tests also increased with age. Therefore women may be at greater risk than men of developing conditions which prevent them from undertaking the test or falling below the threshold above which the test can be performed as they age. As there were no gender differences found in inability to perform the walking test this is unlikely to explain the small changes found in the gender difference in walking speed with age which could be due to chance or to gender and age differences in conditions of the cardiovascular or musculoskeletal system which impact on walking ability. Gender differences in survival could also partially explain the findings in relation to grip strength because the longer life expectancy of women than men could result in greater healthy survivor effects among men than women at older ages.

The heterogeneity in gender differences between HALCyon cohorts that was found in our analyses could, as in any other study of this nature which attempts to draw together data from different sources [74], be due to heterogeneity in sampling, geographical location (while some cohorts are drawn from across the UK, many are from specific geographic regions which may differ from others in influential ways), birth cohort and instruments and methods of measuring physical capability, some of which are addressed in further detail below.

### Methodological considerations

Heterogeneity between studies leads us to be cautious about drawing conclusions about the differences in absolute levels of physical capability across cohorts and is the reason why our analyses focus on within-study comparisons which are then pooled [63]. By harmonising the physical capability measures and performing coordinated analyses we have attempted to minimise the heterogeneity between cohorts due to differences in instruments, methods of measurement and analysis. A limitation of our work is that we did not take account of other differences between cohorts. Although we had planned to examine birth cohort differences in physical capability there were too many other differences between cohorts other than birth year and insufficient variation in birth year at any particular age of measurement to allow us to do this satisfactorily (table S4).

Major strengths of this study are the large sample size, gained by drawing together data from several cohorts, the harmonisation of physical capability data and our coordinated analyses of these data. Although there are other potential indicators of physical capability that could have been investigated had the data been available (such as tests of flexibility and endurance) we were also able to examine a wider range of different objective measures of physical capability than many previous studies. One of the differences in protocol between studies which it was necessary to consider when harmonising data was the use in different studies of varying numbers of trials, for example, in HAS and HCS only one trial of TUG was performed whereas in Boyd Orr and CaPS two trials were performed. In the main analyses, where more than one trial had been performed we used either the best or average measure as appropriate. The measures from different trials within cohorts were highly correlated and sensitivity analyses showed no differences in findings when analyses were repeated using individual trial, average and best measures (results not shown).

It was also necessary to consider differences in distance and pace walked and type of handgrip dynamometer used when harmonising walking speed and grip strength data, respectively. For example, differences in distance and pace influence walking speed [75]. However, in multivariable analyses pace but not distance was related to the mean speed recorded and intra-individual variability in walking speed was not affected by pace [71], [75]. Further, similar patterns of age-related change in walking speed have been shown whether speeds recorded at the normal or fastest pace were used [65]. This suggests that while caution is required when comparing mean levels of walking speed between studies which have instructed study participants to walk at different paces (as in our study when comparing LBC1921 with other cohorts), it is reasonable to combine regression coefficients from tests of association from studies which have used fastest and normal pace in meta-analyses. Similar conclusions can be made for grip strength based on findings from previous studies comparing different dynamometers [76], [77].

Although one Canadian study of TUG reported only moderate test-retest reliability [78], the majority of studies have shown that TUG and the other four objective measures of physical capability examined in this study have high levels of reliability [36], [40], [42], [60], [71], [75]–[77], [79], [80]. A major strength of our analyses is the use of objective measures of physical capability which allow us to examine variation in function across the full spectrum of ability. However, in many cases the use of these measures results in the exclusion of people unable to perform the tests. As it is expected that those people unable to perform the tests would have lower levels of capability and, inability to perform the tests increases with age (table S3) the exclusion of these people from analyses is likely to have led to an underestimation of the size of the age differences in physical capability. It is difficult to compare the prevalence of inability to perform the tests across cohorts and fully assess the impact of the exclusion of people unable as this information has not been recorded in comparable ways across cohorts, which highlights the need to ensure that in future data collections this information is always recorded in a standardised, detailed way. By using a binary categorisation of standing balance, which was necessary for comparability across studies but which limited our ability to examine variation across the full spectrum of ability of this test, we were able to include people unable to perform the test in our analyses of this measure. Further, most TUG and walking speed protocols allowed people with walking aids, who we found to have slower average speeds than people who did not use a walking aid (results not shown), to participate. Evidence suggests that use of walking aids does not introduce measurement bias [81] and by allowing people to use their aids very few people were excluded as a result of being unable to perform these tests in the majority of studies.

These harmonised data are now being used within HALCyon in longitudinal analyses which examine factors across life that influence physical capability and other ageing phenotypes [82]–[84]. However, we purposefully chose to focus in this paper on cross-sectional analyses as not all HALCyon studies have repeat measures of physical capability as required to examine longitudinal changes with age. While it has been suggested that the use of cross-sectional data could lead to an underestimation of the size of age-related changes in physical capability [45], [85], studies that have compared findings from cross-sectional and longitudinal analyses have found that they concur [32], [86].

### Conclusions

This collaborative study of British men and women aged 50-90+ years has shown that there are common patterns of age and gender differences in physical capability levels. These analyses provide the foundation for work in progress to investigate lifetime social and biological determinants of physical capability and its changes with age. This cross-cohort study highlights the work required to undertake large collaborations and harmonise data and demonstrates that even where different studies appear to have performed a similar battery of tests, there are variations in protocol which need to be given full consideration. However, harmonisation is possible and in the future, further research may be facilitated by using age-specific standardised protocols for tests of physical capability across these and other cohorts.

## Supporting Information

Figure S1 **Periods of prospective data collection in each of the HALCyon cohorts.** Footnote: not to scale; This table represents only that data which was collected prospectively but additional information on factors earlier in life was ascertained retrospectively in later life in many studies. HAS Hertfordshire Ageing Study; HCS Hertfordshire Cohort Study; CaPS Caerphilly Prospective Study; ELSA English Longitudinal Study of Ageing; NSHD National Survey of Health and Development; NCDS National Child Development Study. SMS – Scottish Mental Survey administered at age 11 y.
(TIFF)

Table S1 **Description of the sample in each of the HALCyon cohorts.** Note: LBC1921  =  Lothian Birth Cohort 1921; HAS  =  Hertfordshire Ageing Study; HCS  =  Hertfordshire Cohort Study; CaPS  =  Caerphilly Prospective Study; ABC1936  =  Aberdeen Birth Cohort 1936; ELSA  =  English Longitudinal Study of Ageing; NSHD  =  MRC National Survey of Health and Development (1946 British birth cohort); NCDS  =  National Child Development Study (1958 British birth cohort). For other information about the HALCyon cohorts please see table 1.
(DOC)

Table S2 **Details of measures of physical capability available in each of the HALCyon cohorts.** Note: TUG = Timed get up and go. LBC1921  =  Lothian Birth Cohort 1921; HAS  =  Hertfordshire Ageing Study; HCS  =  Hertfordshire Cohort Study; CaPS  =  Caerphilly Prospective Study; ABC1936  =  Aberdeen Birth Cohort 1936; ELSA  =  English Longitudinal Study of Ageing; NSHD  =  MRC National Survey of Health and Development (1946 British birth cohort); NCDS  =  National Child Development Study (1958 British birth cohort).
(DOC)

Table S3 **Age and gender distribution of those people in each cohort who were coded as being unable to perform each of the tests of physical capability.** Note: LBC1921 not shown as information on those unable to perform the tests was not available. Information on standing balance not shown as those people who were unable to perform the test were included in analyses (coded as unable to balance for at least 5 seconds). For chair rises ‘unable’ includes those people who were unable to attempt the test and also those people who were unable to successfully complete the test. LBC1921  =  Lothian Birth Cohort 1921; HAS  =  Hertfordshire Ageing Study; HCS  =  Hertfordshire Cohort Study; CaPS  =  Caerphilly Prospective Study; ABC1936  =  Aberdeen Birth Cohort 1936; ELSA  =  English Longitudinal Study of Ageing; NSHD  =  MRC National Survey of Health and Development (1946 British birth cohort); NCDS  =  National Child Development Study (1958 British birth cohort).
(DOC)

Table S4 **Birth years by age group in each HALCyon cohort.** a: 53 y only in NSHD; 52–54 y in ELSA; b: 59 y only in HCS; c: 63–64 y in HAS and Boyd Orr; 62–64 y in ABC1936 and in HCS for chair rises and standing balance; 61–64 y in HCS for walking speed and timed get up and go; d: 65–68 y in ABC1936; e: 70–73 y in both HAS and HCS for grip strength; 72–74 y in HAS for chair rises, walking speed, timed get up and go and standing balance; f: 77–80 y in LBC1921; g: 80–83 y in HAS; 80–82 y in Boyd Orr. Note: LBC1921  =  Lothian Birth Cohort 1921; HAS  =  Hertfordshire Ageing Study; HCS  =  Hertfordshire Cohort Study; CaPS  =  Caerphilly Prospective Study; ABC1936  =  Aberdeen Birth Cohort 1936; ELSA  =  English Longitudinal Study of Ageing; NSHD  =  MRC National Survey of Health and Development (1946 British birth cohort); NCDS  =  National Child Development Study (1958 British birth cohort).
(DOC)
