# Supplementary material for: Age and Gender Differences in Physical Capability Levels from Mid-Life Onwards: The Harmonisation and Meta-Analysis of Data from Eight UK Cohort Studies
Source: PLoS One. 2011 Nov 16;6(11):e27899. doi: 10.1371/journal.pone.0027899 (PMC3218057; doi:10.1371/journal.pone.0027899)
Supplement: Table S1 — Description of the sample in each of the HALCyon cohorts. Note: LBC1921 = Lothian Birth Cohort 1921; HAS = Hertfordshire Ageing Study; HCS = Hertfordshire Cohort Study; CaPS = Caerphilly Prospective Study; ABC1936 = Aberdeen Birth Cohort 1936; ELSA = English Longitudinal Study of Ageing; NSHD = MRC National Survey of Health and Development (1946 British birth cohort); NCDS = National Child Development Study (1958 British birth cohort). For other information about the HALCyon cohorts please see table 1. (DOC) [file pone.0027899.s002.doc]

**Table S1: Description of the sample in each of the HALCyon cohorts**

|  | **LBC1921** | **HAS** | **HCS** | **CaPS** | **Boyd Orr** | **ABC1936** | **ELSA** | **NSHD** | **NCDS** |
| --- | --- | --- | --- | --- | --- | --- | --- | --- | --- |
| **Description of sample** | Participants of the 1932 Scottish Mental Survey, still resident in the Edinburgh area of Scotland in 1999 | People born in North Hertfordshire, England between 1920 and 1930 whose birth and infant records were available and who were alive and living in N. Herts in 1994-5 | People born in E, N and West Hertfordshire, England between 1931 and 1939 whose birth and infant records were available and who were alive and living in Herts in 1998 | A random sample of men living in Caerphilly, Wales and adjacent villages, recruited between 1979 and 1983 when aged 45-59 | Participants of the Carnegie Survey of Diet and Health (1937-39), who were traced using the NHS central register | Participants of the 1947 Scottish Mental Survey, still resident in the Aberdeen area of Scotland in 1999 | Nationally representative sample of people aged 50 and over living in private households in England in 2002 (wave 1) who had previously participated in the Health Survey for England in 1998, 1999 or 2001 (wave 0) | Nationally representative sample from England, Scotland and Wales born in March 1946 and followed prospectively since | Nationally representative sample from England, Scotland and Wales born in March 1958 and followed prospectively since |

Note:

LBC1921 = Lothian Birth Cohort 1921; HAS = Hertfordshire Ageing Study; HCS = Hertfordshire Cohort Study; CaPS = Caerphilly Prospective Study; ABC1936 = Aberdeen Birth Cohort 1936; ELSA = English Longitudinal Study of Ageing; NSHD = MRC National Survey of Health and Development (1946 British birth cohort); NCDS = National Child Development Study (1958 British birth cohort)

For other information about the HALCyon cohorts please see table 1.
