# Supplementary material for: Age and Gender Differences in Physical Capability Levels from Mid-Life Onwards: The Harmonisation and Meta-Analysis of Data from Eight UK Cohort Studies
Source: PLoS One. 2011 Nov 16;6(11):e27899. doi: 10.1371/journal.pone.0027899 (PMC3218057; doi:10.1371/journal.pone.0027899)
Supplement: Table S2 — Details of measures of physical capability available in each of the HALCyon cohorts. Note: TUG = Timed get up and go. LBC1921 = Lothian Birth Cohort 1921; HAS = Hertfordshire Ageing Study; HCS = Hertfordshire Cohort Study; CaPS = Caerphilly Prospective Study; ABC1936 = Aberdeen Birth Cohort 1936; ELSA = English Longitudinal Study of Ageing; NSHD = MRC National Survey of Health and Development (1946 British birth cohort); NCDS = National Child Development Study (1958 British birth cohort). (DOC) [file pone.0027899.s003.doc]

**Table S2: Details of measures of physical capability available in each of the HALCyon cohorts**

| **Cohort** | **Grip strength** | **Chair rises** | **Walking speed & TUG** | **Standing balance** |
| --- | --- | --- | --- | --- |
| **LBC1921** | 3 measures from the dominant hand using a Jamar or North Coast hydraulic hand dynamometer | Not assessed | Time to walk 6m from a standing start as quickly but as safely as possible | Not assessed |
|  |  |  |  |  |
| **HAS** | 6 measures (3 from dominant hand, 3 from non-dominant hand) using Harpenden handgrip dynamometer at first age (63-73) and Jamar hydraulic dynamometer at second age (72-83) | Time taken to rise from a sitting to a standing position and sit down again 5 times, as fast as possible | Walking speed:Time to walk 3m from a standing start at a normal pace  Timed get up and go: time to get up from a chair, walk 3m at a normal pace turn around, return to the chair and sit back down again | The longest time up to 30s that a one-legged stance could be maintained (performed once with eyes open) |
|  |  |  |  |  |
| **HCS** | 6 measures (3 from dominant hand, 3 from non-dominant hand) using Jamar hydraulic dynamometer (measured at both waves, but second wave only included sub-sample of first wave) | Time taken to rise from a sitting to a standing position and sit down again 5 times, as fast as possible | Walking speed:Time to walk 3m from a standing start at a normal pace  Timed get up and go: time to get up from a chair, walk 3m at a normal pace turn around, return to the chair and sit back down again | The longest time up to 30s that a one-legged stance could be maintained (performed once with eyes open) |
|  |  |  |  |  |
| **CaPS** | Not assessed | Not assessed | Walking speed not assessed but 2 trials of timed get up and go performed (time to get up from a chair, walk 3m at a normal pace turn around, return to the chair and sit back down again) | The longest time up to 30s that a one-legged stance could be maintained (performed twice with eyes open) |
|  |  |  |  |  |
| **Boyd Orr** | Not assessed | Not assessed | Walking speed not assessed but 2 trials of timed get up and go performed (time to get up from a chair, walk 3m at a normal pace turn around, return to the chair and sit back down again) | The longest time up to 30s that a one-legged stance could be maintained (performed twice with eyes open) |
|  |  |  |  |  |
| **ABC1936** | Not assessed | Assessed ability to stand from a sitting position but did not measure time to rise from a chair a specified number of times | Time to walk 6m from a standing start at a normal pace | Ability to balance on one leg with eyes open for 5s |
|  |  |  |  |  |
| **ELSA** | 6 measures (3 from dominant hand, 3 from non-dominant hand) using a Smedley’s handgrip dynamometer | Time taken to rise from a sitting to a standing position and sit down again 5 times, as fast as possible | Time to walk 8 feet from a standing start at a normal pace (among those participants aged 60+) | Times up to a maximum of 10s that a side by side, semi-tandem and tandem stand could be maintained, with only those participants able to perform a stand for 10s asked to perform the subsequent stand in the sequence (all those 69 and under who completed all three tandem stands successfully were also timed standing on one leg with eyes open for up to 30s) |
|  |  |  |  |  |
| **NSHD** | 4 measures (2 from dominant hand, 2 from non-dominant hand) using an electronic handgrip dynamometer | Time taken to rise from a sitting to a standing position and sit down again 10 times, as fast as possible | Not assessed | The longest time up to 30s that a one-legged stance could be maintained (performed once with eyes open and once with eyes closed) (time for eyes open used in analyses presented) |
|  |  |  |  |  |
| **NCDS** | Not assessed | Not assessed | Not assessed | Not assessed |

Note: TUG=Timed get up and go

LBC1921 = Lothian Birth Cohort 1921; HAS = Hertfordshire Ageing Study; HCS = Hertfordshire Cohort Study; CaPS = Caerphilly Prospective Study; ABC1936 = Aberdeen Birth Cohort 1936; ELSA = English Longitudinal Study of Ageing; NSHD = MRC National Survey of Health and Development (1946 British birth cohort); NCDS = National Child Development Study (1958 British birth cohort)
