# Supplementary material for: Age and Gender Differences in Physical Capability Levels from Mid-Life Onwards: The Harmonisation and Meta-Analysis of Data from Eight UK Cohort Studies
Source: PLoS One. 2011 Nov 16;6(11):e27899. doi: 10.1371/journal.pone.0027899 (PMC3218057; doi:10.1371/journal.pone.0027899)
Supplement: Table S3 — Age and gender distribution of those people in each cohort who were coded as being unable to perform each of the tests of physical capability. Note: LBC1921 not shown as information on those unable to perform the tests was not available. Information on standing balance not shown as those people who were unable to perform the test were included in analyses (coded as unable to balance for at least 5 seconds). For chair rises ‘unable’ includes those people who were unable to attempt the test and also those people who were unable to successfully complete the test. LBC1921 = Lothian Birth Cohort 1921; HAS = Hertfordshire Ageing Study; HCS = Hertfordshire Cohort Study; CaPS = Caerphilly Prospective Study; ABC1936 = Aberdeen Birth Cohort 1936; ELSA = English Longitudinal Study of Ageing; NSHD = MRC National Survey of Health and Development (1946 British birth cohort); NCDS = National Child Development Study (1958 British birth cohort). (DOC) [file pone.0027899.s004.doc]

**Table S3: Age and gender distribution of those people in each cohort who were coded as being unable to perform each of the tests of physical capability**

| **Cohort** | **Grip strength** | **Chair rises** | **Walking and TUG speed** |
| --- | --- | --- | --- |
| **HAS** | No reasons recorded for missing data. | - 22 (7.5 %) unable  - Men: 6.4%; Women; 9.1% (p=0.38)  - 72-74y: 7.5%; 75-79y: 6.4%; 80-83y: 23.1% (p=0.09) | - 1 (0.3%) unable  - Cannot assess age and gender differences |
|  |  |  |  |
| **HCS** | No reasons recorded for missing data. | - 56 people (3.4 %) unable  - Men: 3.1%; Women; 3.6% (p=0.65)  - 62-64y: 3.1%; 65-69y: 3.3%; 70-74y: 3.6% (p=0.92) | - 3 (0.1%) unable  - Cannot assess age and gender differences |
|  |  |  |  |
| **CaPS** | Not assessed | Not assessed | - 18 (1.6%) unable  - N/A - Men only study  - 65-69y: 1.6%; 70-74y: 0.9%; 75-79y: 1.9%; 80+y: 3.8% (p=0.28) |
|  |  |  |  |
| **Boyd Orr** | Not assessed | Not assessed | - 1 (0.3%) unable  - Cannot assess age and gender differences |
|  |  |  |  |
| **ABC1936** | Not assessed | Measure not included | - 2 (0.4%) unable  - Cannot assess age and gender differences |
|  |  |  |  |
| **ELSA** | - 63 (0.8%) unable  - Men: 0.4%; Women: 1.2% (p=0.001)  - 52-54y: 0%; 55-59y: 0.4%; 60-64y: 0.4%; 65-69y: 0.6%; 70-74y: 1.1%; 75-79y: 1.1%; 80-84y: 2.4%; 85-89y: 2.9%; 90+y: 5.1% (p<0.01) | - 715 (10.1%) unable  - Men: 9.0%; Women: 11.1% (p=0.004)  - 52-54y: 3.7%; 55-59y: 5.5%; 60-64y: 7.1%; 65-69y: 7.8%; 70-74y: 9.4%; 75-79y: 13.2%; 80-84y: 28.2%; 85-89y: 33.5%; 90+y: 50.8% (p<0.01) | - 425 (7.2%) unable  - Men: 6.7%; Women: 7.6% (p=0.17)  - 60-64y: 4.8%; 65-69y: 4.2%; 70-74y: 6.9%; 75-79y: 6.9%; 80-84y: 12.8%; 85-89y: 16.8%; 90+y: 22.1% (p<0.01) |
|  |  |  |  |
| **NSHD** | - 69 (2.4%) unable  - Men: 2.1%; Women: 2.6% (p=0.34)  - N/A – cohort all same age | - 154 (5.3%) unable  - Men: 5.0%; Women: 5.6% (p=0.45)  - N/A – cohort all same age | Not assessed |
|  | **Each cell of the table represents:**  **- Total no. (%) unable to undertake the specified test**  **- % of men and women unable (p-value from chi-squared test of gender difference)**  **- % unable in each age category (p-value from chi-squared test of age difference)** | | |

Note: LBC1921 not shown as information on those unable to perform the tests was not available

Information on standing balance not shown as those people who were unable to perform the test were included in analyses (coded as unable to balance for at least 5 seconds)

For chair rises ‘unable’ includes those people who were unable to attempt the test and also those people who were unable to successfully complete the test

LBC1921 = Lothian Birth Cohort 1921; HAS = Hertfordshire Ageing Study; HCS = Hertfordshire Cohort Study; CaPS = Caerphilly Prospective Study; ABC1936 = Aberdeen Birth Cohort 1936; ELSA = English Longitudinal Study of Ageing; NSHD = MRC National Survey of Health and Development (1946 British birth cohort); NCDS = National Child Development Study (1958 British birth cohort)
