# Supplementary material for: Age and Gender Differences in Physical Capability Levels from Mid-Life Onwards: The Harmonisation and Meta-Analysis of Data from Eight UK Cohort Studies
Source: PLoS One. 2011 Nov 16;6(11):e27899. doi: 10.1371/journal.pone.0027899 (PMC3218057; doi:10.1371/journal.pone.0027899)
Supplement: Table S4 — Birth years by age group in each HALCyon cohort. a: 53 y only in NSHD; 52–54 y in ELSA; b: 59 y only in HCS; c: 63–64 y in HAS and Boyd Orr; 62–64 y in ABC1936 and in HCS for chair rises and standing balance; 61–64 y in HCS for walking speed and timed get up and go; d: 65–68 y in ABC1936; e: 70–73 y in both HAS and HCS for grip strength; 72–74 y in HAS for chair rises, walking speed, timed get up and go and standing balance; f: 77–80 y in LBC1921; g: 80–83 y in HAS; 80–82 y in Boyd Orr. Note: LBC1921 = Lothian Birth Cohort 1921; HAS = Hertfordshire Ageing Study; HCS = Hertfordshire Cohort Study; CaPS = Caerphilly Prospective Study; ABC1936 = Aberdeen Birth Cohort 1936; ELSA = English Longitudinal Study of Ageing; NSHD = MRC National Survey of Health and Development (1946 British birth cohort); NCDS = National Child Development Study (1958 British birth cohort). (DOC) [file pone.0027899.s005.doc]

**Table S4: Birth years by age group in each HALCyon cohort**

|  | **5 yr age groups** | | | | | | | | | |
| --- | --- | --- | --- | --- | --- | --- | --- | --- | --- | --- |
| **Cohort (assessment year/s)** | **50-54^a^** | **55-59^b^** | **60-64^c^** | **65-69^d^** | **70-74^e^** | | **75-79^f^** | **80-84^g^** | **85-89** | **90+** |
| **Grip strength** | | | | | |  | | | | |
| **LBC1921 (1999-2001)** | - | - | - | - | - | | 1921 | - | - | - |
|  |  |  |  |  |  | |  |  |  |  |
| **HAS (1994-1995)** | - | - | 1929-1930 | 1924-1930 | 1920-1925 | | - | - | - | - |
|  |  |  |  |  |  | |  |  |  |  |
| **HCS (1999-2004)** | - | 1939 | 1934-1939 | 1931-1939 | 1931-1934 | | - | - | - | - |
|  |  |  |  |  |  | |  |  |  |  |
| **ELSA (2004)** | 1950-52 | 1945-1949 | 1940-1944 | 1935-1939 | 1930-1934 | | 1925-1929 | 1920-1924 | 1915-1919 | <1914 |
|  |  |  |  |  |  | |  |  |  |  |
| **NSHD (1999)** | 1946 | - | - | - | - | | - | - | - | - |
| **Chair rises** | | | | | |  | | | | |
| **HAS (2003-5)** | - | - | - | - | 1928-1930 | | 1923-1929 | 1921-1924 | - | - |
|  |  |  |  |  |  | |  |  |  |  |
| **HCS (1999-2005)** | - | - | 1937-1939 | 1932-1939 | 1931-1935 | | - | - | - | - |
|  |  |  |  |  |  | |  |  |  |  |
| **ELSA (2004)** | 1950-52 | 1945-1949 | 1940-1944 | 1935-1939 | 1930-1934 | | 1925-1929 | 1920-1924 | 1915-1919 | <1914 |
|  |  |  |  |  |  | |  |  |  |  |
| **NSHD (1999)** | 1946 | - | - | - | - | | - | - | - | - |
| **Walking speed (m/s)** | | | | | |  | | | | |
| **LBC1921 (1999-2001)** | - | - | - | - | - | | 1921 | - | - | - |
|  |  |  |  |  |  | |  |  |  |  |
| **HAS (2003-5)** | - | - | - | - | 1928-1930 | | 1923-1929 | 1921-1924 | - | - |
|  |  |  |  |  |  | |  |  |  |  |
| **HCS (1999-2005)** | - | - | 1936-1939 | 1931-1939 | 1931-1935 | | - | - | - | - |
|  |  |  |  |  |  | |  |  |  |  |
| **ABC1936 (1999-2005)** | - | - | 1936 | 1936 | - | | - | - | - | - |
|  |  |  |  |  |  | |  |  |  |  |
| **ELSA (2004)** | - | - | 1940-1944 | 1935-1939 | 1930-1934 | | 1925-1929 | 1920-1924 | 1915-1919 | <1914 |
| **TUG speed (m/s)** |  | | | | | | | | | |
| **HAS (2003-5)** | - | - | - | - | 1928-1930 | | 1923-1929 | 1921-1924 | - | - |
|  |  |  |  |  |  | |  |  |  |  |
| **HCS (1999-2005)** | - | - | 1936-1939 | 1931-1939 | 1931-1935 | | - | - | - | - |
|  |  |  |  |  |  | |  |  |  |  |
| **CaPS (2002-4)** | - | - | - | 1933-38 | 1928-1934 | | 1922-1929 | 1919-1923 | - | - |
|  |  |  |  |  |  | |  |  |  |  |
| **Boyd Orr (2002-3)** | - | - | 1938-1939 | 1933-1938 | 1928-1933 | | 1923-1928 | 1920-1923 | - | - |
| **Standing balance** | | | | | | | | | | |
| **HAS (2003-5)** | - | - | - | - | 1928-1930 | | 1923-1929 | 1921-1924 | - | - |
|  |  |  |  |  |  | |  |  |  |  |
| **HCS (1999-2005)** | - | - | 1937-1939 | 1932-1939 | 1931-1935 | | - | - | - | - |
|  |  |  |  |  |  | |  |  |  |  |
| **CaPS (2002-4)** | - | - | - | 1933-38 | 1928-1934 | | 1922-1929 | 1919-1923 | - | - |
|  |  |  |  |  |  | |  |  |  |  |
| **Boyd Orr (2002-3)** | - | - | 1938-1939 | 1933-1938 | 1928-1933 | | 1923-1928 | 1920-1923 | - | - |
|  |  |  |  |  |  | |  |  |  |  |
| **ABC1936 (1999-2005)** | - | - | 1936 | 1936 | - | | - | - | - | - |
|  |  |  |  |  |  | |  |  |  |  |
| **ELSA (2004)** | 1950-52 | 1945-1949 | 1940-1944 | 1935-1939 | 1930-1934 | | 1925-1929 | 1920-1924 | 1915-1919 | <1914 |
|  |  |  |  |  |  | |  |  |  |  |
| **NSHD (1999)** | 1946 | - | - | - | - | | - | - | - | - |

a: 53y only in NSHD; 52-54y in ELSA; b: 59y only in HCS; c: 63-64y in HAS and Boyd Orr; 62-64y in ABC1936 and in HCS for chair rises and standing balance; 61-64y in HCS for walking speed and timed get up and go; d: 65-68y in ABC1936; e: 70-73y in both HAS and HCS for grip strength; 72-74y in HAS for chair rises, walking speed, timed get up and go and standing balance; f: 77-80y in LBC1921; g: 80-83y in HAS; 80-82y in Boyd Orr

Note: LBC1921 = Lothian Birth Cohort 1921; HAS = Hertfordshire Ageing Study; HCS = Hertfordshire Cohort Study; CaPS = Caerphilly Prospective Study; ABC1936 = Aberdeen Birth Cohort 1936; ELSA = English Longitudinal Study of Ageing; NSHD = MRC National Survey of Health and Development (1946 British birth cohort); NCDS = National Child Development Study (1958 British birth cohort)
